# Discriminant canonical analysis as a tool to determine traces of endangered native hen breed introgression through egg hatchability phenomics

**DOI:** 10.5713/ab.22.0163

**Published:** 2022-11-14

**Authors:** Antonio González Ariza, Francisco Javier Navas González, Ander Arando Arbulu, José Manuel León Jurado, Juan Vicente Delgado Bermejo, María Esperanza Camacho Vallejo

**Affiliations:** 1 Department of Genetics, Faculty of Veterinary Sciences, University of Córdoba, 14071 Córdoba, Spain; 2 Institute of Agricultural Research and Training (IFAPA), 14004 Córdoba, Spain; 3 Present address: NEIKER – Basque Institute of Agricultural Research and Development, Basque Research and Technology Alliance (BRTA), 01192 Arkaute, Spain; 4 Agropecuary Provincial Centre, Diputación Provincial de Córdoba, 14071 Córdoba, Spain

**Keywords:** Data Mining, Egg Shape and Weight, External Traits, Genetic Resources, Incubation, Native Hen Breeds

## Abstract

**Objective:**

The main objective of this study was to develop a pipeline to detect phenogenomic introgression across different multivariety breeds and to validate such classification focusing on external egg and hatchability-related traits using a discriminant canonical analysis approach.

**Methods:**

For this, 1,368 eggs belonging a flock of 94 endangered Spanish autochthonous breed breeding hens (Andalusian Tufted, Blue Andalusian, Spanish White-Faced, and Utrerana) and a control outgroup comprising 32 eggs belonging to 4 Araucana hens were considered. Multicollinearity analysis of hatchability-related traits revealed embryonic mortality, embryonic mortality in the second stage of incubation, viable hatching chick, major diameter, and minor diameter should be discarded from the analysis (variance inflation factor ≤5) given they did not significantly contribute to variability explanation potential of the discriminant model.

**Results:**

A stepwise discriminant canonical analysis was developed and egg weight, shape index, hatchability, and fertility variables reported the highest discriminant power (Wilks' Lambda values of 0.7861, 0.7871, 0.8076, and 0.9457, respectively). The first two functions explained 85.25% intergroup variability. Interbreed and varieties proximity was evaluated using Mahalanobis distances representation and data mining cross-validation allowed to detect genetic introgression between different genotypes.

**Conclusion:**

Easily collectable traits as egg weight and shape index must be considered for the development of breeding programs as a measure to ensure breed protection. The model may be translatable to other endangered breeds to optimize avian breeds conservation plans worldwide.

## INTRODUCTION

Natural incubation has been discarded from commercial poultry farming models due to its lack of efficiency from an agribusiness perspective. Incubation under natural conditions does not allow working at high production numbers, since a broody bird can only care after a small number of eggs [1]. This situation prompted the development of artificial incubation. Artificial incubation was developed around the idea of replicating a similar environment to natural conditions, based on the control of temperature, humidity, ventilation, and positional movement.

The poultry industry has quantitatively and qualitatively improved in the last decade, which positioned incubation as one of the most determinant areas of development, whose purpose is to produce healthy and excellent quality chicks [2]. Overall hatchability can be defined as the percentage of eggs that, if incubated, eventually produce chicks. Although this productive feature is highly influenced by nutritional, management, and health factors in the breeding flock, as well as by conditions in the incubation process in the hatchery plant it has been ascribed to a hereditary component. Hatchability is in turn influenced by other concepts, such as fertility, embryonic mortality, and even hatching mortality. Furthermore, mortality during the chick's first 5 days of life is related to the quality of the embryo and its viability [3].

Eggs are laid at 42°C, then collected, taken to the hatching plant, and selected depending upon their quality. During this time, they adapt to ambient temperature which ranges from 19°C to 27°C. The exposure of eggs to temperature conditions at the cold room determines the stop of embryonic growth, and a state of lethargy is maintained from 2 to a maximum of 15 days (optimal time of 4 days for chicken eggs) [4].

Significant differences in reproductive traits have been reported when different native breeds are compared. The stability of the diversity of reproductive traits, as well as the genetic potential of these birds allows native avian genetic resources to be regarded as an important pool of genes contributing to the global biodiversity. In this context, poultry production under alternative systems promotes and sets its basis on the use of local hen breeds, which have been selected to be perfectly adapted to local ecosystems. This allows the production of differentiated food (eggs and meat) under adverse management and weather conditions [5,6]. Contextually, Andalusia, located in southern Spain, is influenced by Mediterranean climate, with very high temperatures from May to October, according to the Spanish State Meteorological Agency (AEMET).

Regional breeders have traditionally centered their activity on the use of the Mediterranean avian trunk, which comprises the genotypes studied in the present work (Andalusian Tufted, Blue Andalusian, Spanish White-Faced, and Utrerana). Generally, these breeds have shared the same geographic area and a common phylogenetic connection and have traditionally been bred in backyard systems and extensive conditions [7]. All these breeds are characterized by lighter individuals with white earlobes, which lay white-shelled eggs.

Several studies have focused on disentangling the existing genetic, morphological, productive, and reproductive differences across Andalusian autochthonous hen breeds. In this research framework, discriminant canonical analysis has been used to develop statistically validated pipelines for egg quality and morphological classification [6,8]. Although discriminant analysis has been deemed very useful to reveal data clustering patterns, the efficiency of these statistical approaches may be reinforced by the complementarity that exists with new emerging statistical pipelines, which enable determining the specific causes for which a certain individual may ascribe to one cluster instead of others.

In these regards, the collection of large amounts of information prompted large data number processing strategies to emerge to boost the possibilities of precision farming. For instance, expert systems involving data mining [9] allow the extraction of knowledge out of field data and statistically transcribe the logical rules that these data follow. Afterwards, this can be used for the construction of decision trees, which in turn complement and give an explanation to discriminant analysis clustering patterns. Data mining has been successfully applied in different fields of poultry production, as an egg quality predictor, selection of laying breeders, biometric characterization, and house environment suitability [6,8].

Many benefits have been ascribed to the application of data mining techniques. For example, by applying data mining to organize and analyze a sizable set of data, solutions can be obtained faster than traditional methods with optimal predictive performance, which is necessary in large scale studies. Furthermore, missing and noisy data can be better tolerated if numerical, categorical, and date data are correctly combined than when using other statistical techniques. Consequently, efficiency is increased, and potential quality problems can be detected [10].

To this aim, the present study seeks to determine the differential clustering patterns of reproductive traits in data from eggs laid by four Spanish native breeds and their varieties: Andalusian Tufted (Black and White), Blue Andalusian, Spanish White-Faced, and Utrerana (Black, Franciscan, Partridge, and White) when compared to Araucana breed as a foreign native breed outgroup (American continent). This work verifies the benefits that derive from the synergy of discriminant canonical analysis and data mining through the development of a functional pipeline to quantify the historical introgression of these native breeds and varieties. The outcomes of the present study may support the characterization of the Spanish laying hen breeds, as a strategy to support the conservation and sustainability of the breeding program of endangered genotypes.

## MATERIALS AND METHODS

### Ethical approval

Protocols applied were permitted by the regulations of the European Union (2010/63/EU) in their transposition to the Royal Decree-Law 53/2013. No experimental procedure has been performed in the present study, since all the data were obtained in the framework of normal hatchery activity. Therefore, the present study is out of the scope of evaluation of the Ethics review board of the University of Córdoba, as it does not fall under the legislation for the protection of animals used for scientific purposes.

### Animals, sample size, and background

The experiment was performed at the Agropecuary Provincial Center (Diputación Provincial de Córdoba) in Córdoba (37°54′50.9″N to 4°42′40.4″W, Andalusia, southern Spain). Data were collected during the breeding season (February to April 2019). A total of 98 laying hens (Table 1) were selected as the experimental breeding group. The flock of layers, with an average age of 40.44±19.20 weeks, were placed in pens with 50% of their surface uncovered and natural lighting (stocking density: 1 hen per m2). All the breeding groups were fed with the same commercial feed (chemical composition: 15.20% crude protein, 14.00% crude ashes, 4.60% crude fats and oil, 4.10% calcium, 3.20% crude fiber, 0.72% lysine, 0.66% phosphorus, 0.31% methionine, 0.19% sodium). Animals had free access to water and food *ad libitum*. All hens were fertilized by a rooster belonging to their same genotype through directed natural mating every 5 days. One rooster was used for every 4 or 5 hens. All roosters were reproductively tested to ensure their optimal suitability for mating.

A total of 1,400 eggs was collected during the study (Table 1). All eggs were daily collected, identified, and stored under optimal conditions (temperature [T^a^]: 17°C to 18°C; 72% to 78% relative humidity) until their incubation.

### External egg measurements

For each egg, the following measurements were taken:

• Egg weight: using an electronic scale (CSB-600C; Cobos, Barcelona, Spain; precision: ±0.01 g).
• Eggshell L*, eggshell a*, and eggshell b*: the eggshell color was determined using a portable spectrophotometer (CM 700d; Konica Minolta Holdings Inc., Tokyo, Japan). The color results were expressed according to the International Commission on Illumination (CIE) L*a*b* system color profile. The coordinates of this system represent the lightness of color (L* = 0 yields black and L* = 100 indicates diffuse white), its position between red and green (a*, negative values indicate green while positive values indicate red) and its position between yellow and blue (b*, negative values indicate blue and positive values indicate yellow).
• Major and minor diameter: a digital caliper (M 60.205; Electro DH, Barcelona, Spain; precision: ±0.01 mm) was used to determine these two measurements, also known as equatorial and polar diameters.
• Shape index (SI): was computed using the following formula [8]:

$$\text{SI}=\frac{\emptyset \text{M}}{\emptyset \text{m}}\times 100$$

Where ØM and Øm are major and minor diameters.

### Incubation conditions and control of hatchability results

The eggs were sorted into 10 incubation batches considering the week in which they were laid. All eggs were externally inspected with the aid of a live candling device (LED; Masalles, Barcelona, Spain) to verify each eggshell was intact and suitable for incubation. The incubation of eggs was performed in an incubator (M240-I; Masalles, Spain) at 37.2°C and 55% to 60% RH, with simultaneous auto-turn every 1 h (45°C from the horizontal plane). On the 19th day of incubation, all eggs were placed in a tray at the hatchery cabinet (25-N HLC; Masalles, Spain) maintained at 36.7°C and 60% RH until hatching (two more days).

Eggs fertilization was checked using a live candling device seven days after the start of incubation. To differentiate infertile from very early embryonic death (<2 days of incubation), eggs were broken out and examined.

At hatching, the chicks were sorted per incubation batch, wing-banded to follow an individual traceability, and placed in rearing rooms (stocking density: 5 birds/m^2^) with electric heaters (Copele LGA; Copele, Murcia, Spain) in each room. Commercial feed and water were available *ad libitum*.

Egg evaluation was performed by trained and experienced hatchery staff. The evaluation of eggs was performed during the incubation process. The following measurements were considered.

• Fertility: whether the egg was fertilized or not. Observation made 7 days after the start of incubation. Eggs which were not fertilized were extracted from the incubator.
• Embryonic mortality: embryo death. All non-hatching eggs were broken at the end of the incubation period and the state of development of the embryo was registered. Dead embryos were sorted depending on the moment when embryo death took place (first -embryonic mortality 1- or second half -embryonic mortality 2- of the incubation period, respectively).
• Hatchability of the egg: integrity of the shell was measured at 21 days from the start of incubation to determine whether hatching had occurred or not.
• Mortality at hatching: chicks that had died at hatching (while loss of eggshell integrity is produced) were counted.
• Viable hatching chicks: whether chick hatched alive.
• Mortality within 5 days after hatching: as a measurement of post-hatch viability. Chicks that had died within 5 days of hatching were scored.

Hatchability traits were measured as dichotomous variables (Yes/No) and named after the following concepts: fertility, embryonic mortality, embryonic mortality 1, embryonic mortality 2, hatchability, mortality at hatching, viable chick, mortality post-hatching.

### Discriminant canonical analysis

This statistical methodology was performed to develop a pipeline that enables the classification of the studied breeds and varieties according to hatchability and external characteristics of their eggs and determine whether a linear combination of these measures describe within and between population clustering group. The explanatory variables used for the present research were egg weight, eggshell L*, eggshell a*, eggshell b*, major diameter, minor diameter, shape index, and presence or absence of fertility, embryonic mortality, embryonic mortality 1, embryonic mortality 2, hatchability, mortality at hatching, viable hatching chick, and mortality post-hatching. The genotype of the breeding hen was considered as the clustering criterion.

A territorial map was constructed through the representation of the canonical trait relationships that were plotted to depict the breed and varieties differences. Regularized forward stepwise multinomial logistic regression algorithms were used to perform the variable selection. Priors were regularized according to the group sizes calculated using the prior probability of a commercial software (IBM SPSS Statistics V.26, for Windows; SPSS, Inc., Armonk, NY, USA) instead of considering them the same, to avoid that groups with different sample sizes affect the quality of the classification.

Settings of the same sample size as the one used in this study across groups have been reported to be robust. A minimum sample size of at least 20 observations for every 4 or 5 predictors, and the maximum number of independent variables of n-2 (where n is the sample size), should be inserted in the analysis so that possible distortion effects are palliated.

In this way, the present study used a relationship between observations and independent variables 4 or 5 times higher than those described above, which causes the sufficient efficiency of the discriminant approaches. Multicollinearity analyses were performed to ensure independence and a strong linear relationship between predictors. The variables chosen by the forward or backward stepwise selection methods were the same. Progressive forward selection method was run, given it is more time efficient than backward selection method.

Discriminant routine of the Classify package of the IBM SPSS Statistics V.26 software and the Discriminant Canonical Analysis routine of the Analyzing Data package of XLSTAT software (Addinsoft Pearson Edition 2014; Addinsoft, Paris, France) were used to perform the discriminant canonical analysis.

#### Multicollinearity preliminary testing

The variance inflation factor (VIF) was computed using a subroutine of the Discriminant Canonical Analysis routine of the Analyzing Data package of the XLSTAT software, as follows:


$$\text{VIF}=\frac{1}{\left(1-{\text{R}}^{2}\right)}$$

where R^2^ is the coefficient of determination of the regression equation.

The most common indicator used in detecting multicollinearity is VIF and a recommended VIF value of 5 was used in this study. The multicollinearity assumption must be tested before running a canonical discriminant analysis, to ensure that redundancies in the variables considered do not condition the structure of the matrices or overinflate the explanatory potential of the variance.

#### Determination of canonical correlation dimension

The maximum number of canonical correlations between two sets of variables is the number of variables in the smaller set. The first canonical correlation generally explains most relationships between different sets. At any rate, all canonical correlations should be considered, even though previous research has reported that only the first dimension is common. When canonical correlation values are ≥0.30, they correspond to about 10% of the variance explained.

#### Discriminant canonical analysis efficiency

Wilks' lambda test was used to evaluate the variables that significantly contribute to the discriminant function. As Wilks's lambda approaches 0, the variable's contribution to the discriminant function increases. The functions can be used to explain group adscription if the significance value is ≤0.05.

#### Discriminant canonical analysis model reliability

Assumption of equal covariance matrices was tested in the discriminant function analysis by Pillai's trace criterion. This is the only acceptable test to be used in cases of unequal sample sizes. It was computed as a subroutine of the Discriminant Canonical Analysis routine of the Analyzing Data package of the XLSTAT software. A significance value of ≤0.05 indicates that the set of predictors considered in the discriminant model being statistically significant; hence, application of Discriminant Canonical Analysis is feasible.

#### Variable dimensionality reduction

A preliminary principal component analysis (PCA) was computed to minimize the overall variables into a few significant variables that contributed most to the different variations in hatchability of eggs. PCA was performed automatically using the Discriminant Analysis routine of the XLSTAT software.

#### Standardized canonical coefficient, loading interpretation, and spatial representation

Discriminant function analysis was used to determine the percentage of allocation of an observation within its group (defined by its breed and variety). Values of ≥|0.40| for the discriminant loading of a variable can be considered to be substantive discriminating variables. It was prevented non-significant variables from the function using a stepwise procedure technique. Large absolute values in coefficients for each variable lead to greater discriminating ability. Data were standardized and squared Mahalanobis distances and PCA were calculated as follows:


$${\text{D}}_{\text{ij}}^{2}=\left({\bar{ϒ}}_{\text{i}}-{\bar{ϒ}}_{\text{j}}\right)\;{\text{COV}}^{-1}\;\left({\bar{ϒ}}_{\text{i}}-{\bar{ϒ}}_{\text{j}}\right)$$

where ${\text{D}}_{\text{ij}}^{2}$ is the distance between population i and j; ϒ̄_i_ and ϒ̄_j_ are the means of the variable x in the ith and jth populations, respectively; and COV^–1^ is the inverse of the covariance matrix of measured variable x.

Euclidean distances matrix was used to convert the squared Mahalanobis distance, and afterward, a dendrogram was depicted using the underweighted pair-group method arithmetic averages (UPGMA; Rovira i Virgili University, Tarragona, Spain), and the Phylogeny procedure of MEGA X V.10.0.5 (Institute of Molecular Evolutionary Genetics, The Pennsylvania State University, State College, PA, USA).

#### Discriminant function cross-validation

The hit ratio parameter can be calculated to determine the probability that an observation of an unknown background is classified correctly in a particular group [8]. The leave-one-out cross-validation option is frequently used by authors to consider for discriminant function validation. Leave-v-out cross-validations are an elaborate and time and resource consuming alternative of cross-validation which involve leaving out all possible subsets of v cases. This does not generally lead to better performance than k-fold (indeed, the leave-one-out cross-validation, occurs when k equals the sample size), given variances are relatively higher (their value changes more for different samples of data than the value for other cross-validation alternatives such as k-fold cross-validation). Consequently, after the configuration of the tree building process was determined by chi-squared tests, a k-fold cross-validation was performed (k = 10). Data was divided into ten subsets of (approximately) equal size. Training was performed 10 times (k), each time excluding one (leaving one out) of the subsets from training but using only the omitted subset to compute error. Hence, a 10-fold cross validation was chosen by means of using every sample record in the training sample and study data to obtain a cross-validated error rate and select an optimal tree that prevents bias and outlier overfitting [6].

Classification accuracy is achieved by the discriminant canonical analysis when the classification rate value is at least 25% higher than that obtained by chance.

These results obtained must be supported by Press′ Q statistic, which is a parameter that is able to compare the discriminating power of the cross-validated function by using the formula:


$$\text{Pres}{\text{s}}^{\prime }\text{Q}={\left[\text{n}-({\text{n}}^{\prime }\text{K})\right]}^{2}/[\text{n }(\text{K}-1)]$$

where n is the number of observations in the sample; n′ is the number of observations correctly classified and K is the number of groups.

The value of Press′ Q statistic should be compared to the critical value of 6.63 for χ^2^ with a degree of freedom in a significance of 0.01. When Press' Q exceeds the critical value of χ^2^ = 6.63, the cross-validated classification can be considered significantly better than chance.

### Data mining CHAID decision tree

Discrete categorized data were classified, predicted, interpreted, and manipulated by the chi-square automatic interaction detection (CHAID) decision tree (DT) data mining method as has been computed in previous studies [6,8]. The CHAID-based algorithm decision support tool includes a root node, branches, and leaf nodes. Each internal node was built around an observation trait (input variables), while a chi-square test significance split criterion (p<0.05) was fulfilled (pre-pruning). For this, it was computed the Tree routine of the Classify package of the IBM SPSS Statistics V.26 software.

## RESULTS

### Discriminant canonical analysis model reliability

Embryonic mortality 2, embryonic mortality, viable chick, major diameter, and minor diameter were the variables discarded from further analyses (VIF values >5; Table 2). The variable discard process for the different traits is shown in Supplementary Table S1.

**Figure 1:**
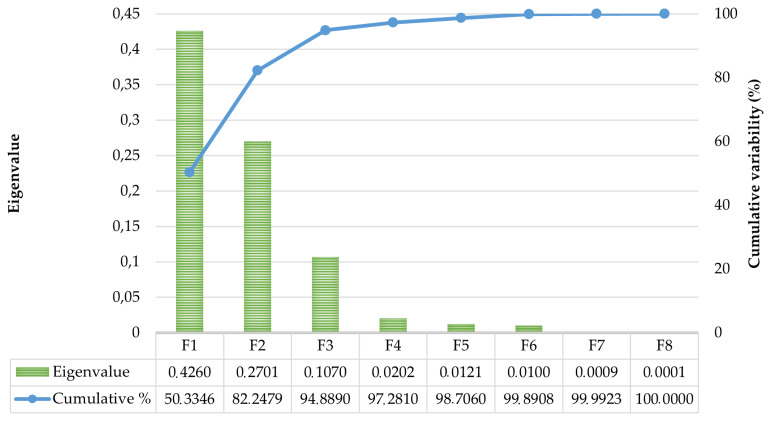
Canonical variable functions and their percentages of self-explained and cumulative variance. The eigenvalue of each discriminant function is used to measure each functions′ discriminative power. The first three functions (F1, F2, and F3) explain 94.89% of variance (Cumulative %).

Significant Pillai's trace criterion (Pillai's trace criterion, 0.6507; df1, 80; df2, 11112; p<0.0001) determined the validity of the discriminant canonical analysis. Significant discriminant abilities were reported for three out of the eight functions revealed after the discriminant analysis as reported in Table 3. The discriminatory power of the F1 function was high (eigenvalue of 0.43; Figure 1) with 94.89% of the variance explained by F1, F2, and F3.

### Standardized canonical coefficients, loading interpretation, and spatial representation

The different variables studied in this research were ranked according to their discriminating ability. A test of equality of group means across hatchability and external characterization traits was used as shown in Table 4. A better discriminating power is indicated by greater values of F and consequently, lower values of Wilks' Lambda. The present analysis revealed that eggshell color (Eggshell L*, eggshell a*, and eggshell b*) did not significantly contribute (p<0.05) to the discriminant functions.

**Figure 2:**
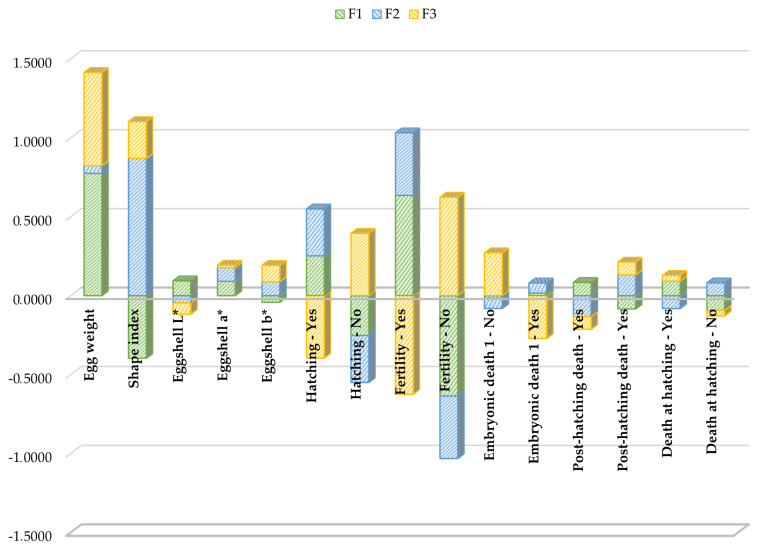
Discriminant coefficients for hatchability and egg external quality-related traits on each canonical discriminant function. Each bar represents the relative weights (loadings) of each particular trait across the three discriminant functions evidenced by the discriminant canonical analysis (F1, F2, and F3: functions 1, 2, and 3).

**Figure 3:**
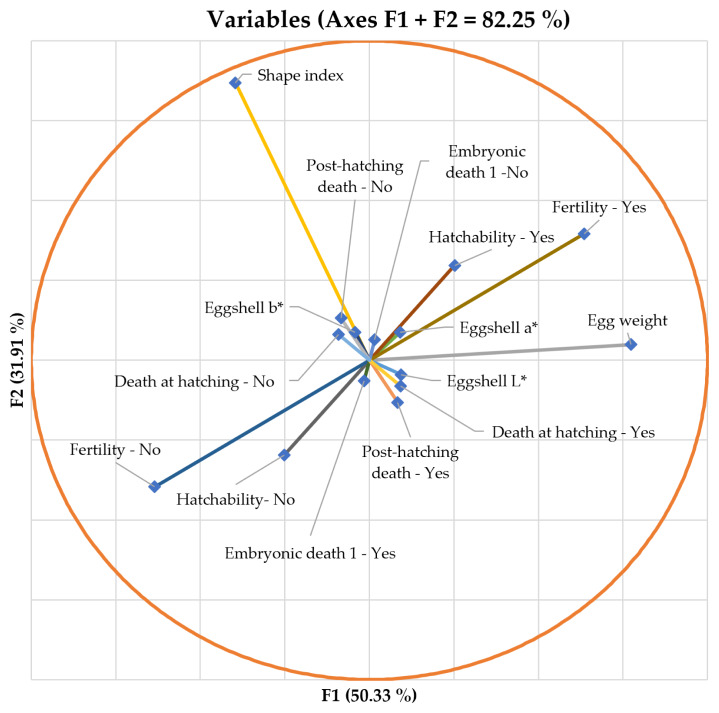
Vector plot for discriminant loadings for the traits considered in discriminant analysis. Those variables which are extended further apart from the origin are the most representatives discriminating ones (positively or negatively) in the two first functions (F1 and F2). Percentages in parentheses represent the variance explanantory potential (%) of each function.

Standardized discriminant coefficients measure the relative weight of each trait across the established discriminant functions (Figures 2 and 3). After discriminant ability testing was performed and depending on the standardized discriminant coefficients of each significant variable, the equation (tool core) for the three functions which significantly explained data variability is as follows:


$$\begin{array}{l} \text{F}1:(0.710)\times \text{Egg Weight}+(-0.301)\times \text{Shape Index}+(0.051)\\ \times \text{Eggshell }{\text{L}}^{*}+(0.141)\times \text{Eggshell }{\text{a}}^{*}+(-0.012)\times \text{Eggshell}\\ {\text{b}}^{*}+(0)\times \text{Hatchability}-\text{Yes}+(0.039)\times \text{Hatchability}-\\ \text{No}+(0)\times \text{Fertility}-\text{Yes}+(-0.604)\times \text{Fertility}-\text{No}+(0.042)\\ \times \text{Embryonic Mortality }1-\text{Yes}+(0)\times \text{Embryonic Mortality}\\ 1-\text{No}+(0.009)\times \text{Post-hatching Mortality}-\text{Yes}+(0)\times \\ \text{Post-hatching Mortality}-\text{No}+(0.043)\times \text{Mortality at}\\ \text{Hatching}-\text{Yes}+(0)\times \text{Mortality at Hatching}-\text{No} \end{array}$$

$$\begin{array}{l} \text{F}2:(0.128)\times \text{Egg Weight}+(0.893)\times \text{Shape Index}+(-0.028)\\ \times \text{Eggshell }{\text{L}}^{*}+(0.025)\times \text{Eggshell }{\text{a}}^{*}+(0.078)\times \text{Eggshell}\\ {\text{b}}^{*}+(0)\times \text{Hatchability}-\text{Yes}+(-0.151)\times \text{Hatchability}-\\ \text{No}+(0)\times \text{Fertility}-\text{Yes}+(-0.372)\times \text{Fertility}-\text{No}+\\ (-0.087)\times \text{Embryonic Mortality }1-\text{Yes}+(0)\times \text{Embryonic}\\ \text{Mortality }1-\text{No}+(-0.146)\times \text{Post-hatching Mortality}-\\ \text{Yes}+(0)\times \text{Post-hatching Mortality}-\text{No}+(-0.002)\times \\ \text{Mortality at Hatching}-\text{Yes}+(0)\times \text{Mortality at Hatching}\\ -\text{No} \end{array}$$

$$\begin{array}{l} \text{F}3:(0.642)\times \text{Egg Weight}+(0.281)\times \text{Shape Index}+(-0.009)\\ \times \text{Eggshell }{\text{L}}^{*}+(-0.055)\times \text{Eggshell }{\text{a}}^{*}+(0.093)\times \text{Eggshell}\\ {\text{b}}^{*}+(0)\times \text{Hatchability}-\text{Yes}+(0.226)\times \text{Hatchability}-\\ \text{No}+(0)\times \text{Fertility}-\text{Yes}+(0.516)\times \text{Fertility}-\text{No}+\\ (0.299)\times \text{Embryonic Mortality }1-\text{Yes}+(0)\times \text{Embryonic}\\ \text{Mortality }1-\text{No}+(-0.071)\times \text{Post-hatching Mortality}-\\ \text{Yes}+(0)\times \text{Post-hatching Mortality}-\text{No}+(0.098)\times \\ \text{Mortality at Hatching}-\text{Yes}+(0)\times \text{Mortality at Hatching}\\ -\text{No} \end{array}$$

**Figure 4:**
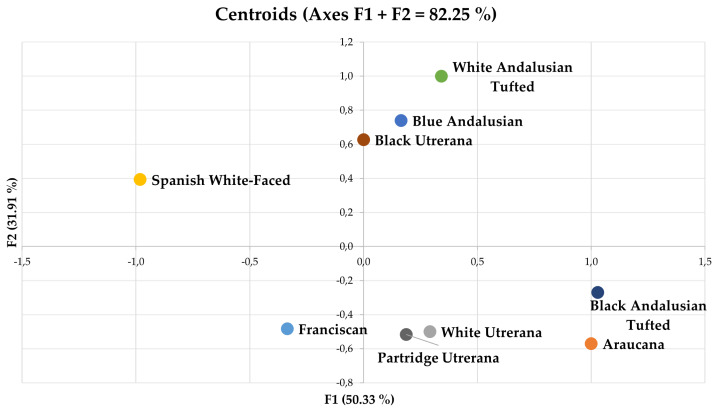
Territorial map depicting the centroids of the different observations considered in the discriminant canonical analysis sorted across breeds and varieties. The relative position of each centroid was determined by substituting the mean value for the observations depicted in the two first discriminant functions (F1 and F2).

In Figure 4, centroids from different breeds and varieties considered in this study are represented. The relative position of each centroid was determined by substituting the mean value for the observations depicted in the two first discriminant functions (F1 and F2). A Press' Q value of 498.13 (n = 1,400; n′ = 418; K = 9) was computed. Thus, predictions can be better than chance at 95%.

**Figure 5:**
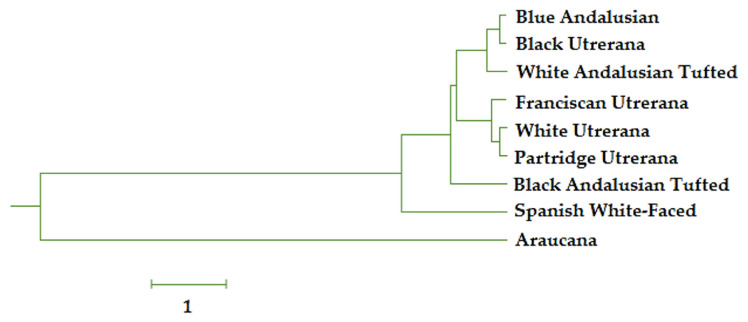
Cladogram constructed from Mahalanobis distances between breeding hen genotype pairs. Mahalanobis distances obtained after the evaluation of the discriminant analysis matrix were transformed into squared Euclidean distances. This determines the similarity between the data registries belonging to each genotype.

Mahalanobis distances across genotypes and varieties were represented in a cladogram (Figure 5). Araucana breed was the most distant breed when compared to the representative breeds of the Mediterranean hen trunk. A close connection between Blue Andalusian, Black Utrerana, and White Andalusian Tufted was evidenced. Still, a central Utrerana egg cluster, with a significantly closer relationship between Franciscan, White, and Partridge varieties was reported. Last, slight similarities were reported between Black Andalusian Tufted and Spanish White-Faced individuals.

### Data mining CHAID decision tree

The underlying basis for these classification patterns was represented in Supplementary Figure S1, after the evaluation of the data mining CHAID decision tree obtained of chi-square dissimilarity matrix. In these regards, Chi-squared based branch, and node distribution suggested observations significantly (p<0.05) differed, thus, were classified in seven groups depending on their values of shape index (≤72.31; 72.31 to 73.09; 73.09 to 73.79; 73.79 to 74.55; 74.55 to 75.30; 75.30 to 77.58; >77.58). After this distinction was revealed, observations were sorted into subgroups depending on egg shape and hatchability variables.

Cross-validation supported the robustness of the results obtained and the validity of the conclusions drawn from them. Similar values of resubstitution (probability or misclassifying an unseen instance) and cross-validation error rate estimates were obtained (0.676 and 0.724, respectively) in the data mining decision tree tenfold cross-validation. The classifier error can be underestimated by data resubstitution. However, it has a lower variability when compared with other methods, such as cross-validation, especially when working with small sample sizes. In this way, the cross-validation error rate estimates were close to but lower than, the replacement estimates. Therefore, the trees were not overfitted, confirming the robustness of the results obtained. Additionally, the number of erroneously classified observations for each genotype was computed, that is the number of individuals which could presumably be ascribed to other genotypes. The outputs of the evaluation of prior and posterior classification of observations presented in Supplementary Table S2, enabled the development of a pipeline to infer the degree introgression across genotypes.

## DISCUSSION

This study analysed a combination of egg external measurements and reproductive parameters of egg batches from a conservation center of native breeds. Verifying the relationships across explanatory variables, selecting those independent variables which do not overlap in terms of their ability to explain data variability is compulsory when deciding on which factors comprise an effective predictive model [6,8,10]. Variables for which multicollinearity problems were evidenced (VIF>5) were not considered further in the analyses. Strong correlations between major and minor diameter with shape index can be mathematically explained by the fact that these terms are included in the mathematical expression used for shape index calculation. In addition, viable chick, embryonic mortality, and embryonic mortality 1 were also deemed redundant since embryonic mortality was computed from the sum of embryonic mortality 1 and embryonic mortality 2. Moreover, the event of an egg hatching (described by the variable hatchability) categorically means no embryonic mortality has occurred as well. The number of viable chicks is also very similar to the number of hatched eggs, hence supporting the basis for these variables being strongly correlated.

The lowest discriminating power was reported for egg-shell color traits (eggshell L*, eggshell a*, and eggshell b*) (Table 4). Contextually, Taha [11] reported that eggshell color has a slight effect on fertility and no effect on hatchability and embryonic mortality from day 8 to 14 of incubation in Japanese quails and Eleroğlu et al [12] obtained similar results since fertility and hatchability were not significantly influenced by eggshell color in eggs from Guinea fowl. The present paper is the first evidence of this slight effect in hen breeds. In any case, all the studied Mediterranean breeds (Blue Andalusian, Andalusian Tufted, Utrerana, and Spanish White Faced) lay white shell eggs. Only the Araucana hen, which lays green-blue shell eggs, was significantly distant from the rest of the genotypes when Mahalanobis distances were represented in the cladogram, and this finding could partly be ascribed to eggshell color coordinates.

Embryonic mortality 1, mortality at hatching, and post-hatching mortality 1 were ranked 5th, 6th, and 7th positions (Table 4). Many factors can affect early embryonic mortality. Among them, egg storage and the genotype and age of breeding hen since albumen quality can be strongly conditioned by these parameters [7,13]. If albumen quality declines, CO2 may escape too quickly which in turn changes the acid-base balance of the embryo causing its death [3]. Commercial hybrid lines have been reported to present a lower quality of albumen when compared with local breeds [14]. Thus, it may be indicative that the selection of highly productive animals may indeed collaterally affect many other factors, for example, may parallelly translate into a decrease in reproductive parameters.

On the other hand, many reports have addressed chromosomal abnormalities to be potentially responsible for 1% to 12% deaths during early embryonic development stages [15]. In this regard, the detrimental effects of numerical chromosomal alterations such as triploidy, haploidy, and polyploidy have been reported to occur at the beginning of embryonic development, as well as in the meiosis or fertilization processes [16]. The first hours and days of life of chicks are a sensitive period during which most of the chicks' organs are still immature [17].

Many factors have been reported to significantly affect mortality at hatching and post-hatching mortality in chicks, such as chick genotype, breeder flock age, egg storage time, and chick gender [3,18]. Early mortality is a very important parameter because it is a measure of egg and chick quality and influences the price of the chicks. Thus, chick mortality is used as an indicator of welfare. Literature reports that some hen genotypes are prone to higher post-hatching mortality due to genetic factors and that mortality rates could be maintained throughout the following stages of the chicks' growing period [18]. For these reasons, breed-related factors and their influence on chick quality should be considered by hatchery managers before selecting the genotype which best fits each type of production system on each case.

From a population genetics point of view, native bird populations represent a pool of genes which are the basis for the better resilience of these breeds in their local environments. These genes encode for traits such as disease resistance, anti-predation behavior, and foraging ability [6], and have been reported to be negatively correlated to productive traits. Therefore, when a group of genes is introduced into a population that increases the productivity of individuals (towards meat or egg-laying aptitude), the negative correlation with the rest of the factors causes the loss of economic viability of farms with non-intensive conditions. This primarily occurs because the natural selection deviates from the better ability towards camouflage of chick colors, and anti-predator or foraging behaviors [19] to the artificial selection focus for better productive features.

Fertility and hatchability, as well as other reproductive traits, characterize by low and stable heritability values (h^2^<0.2) [20]. However, still significant differences in fertility and hatchability traits across breeds or genotypes occur, and may base upon a widely variable physiological range of components [21].

In this context, the storage of sperm in the female reproductive tract requires an adequate microenvironment. The biological mechanisms regulating sperm selection, for instance, a superabundance of secretion of fatty acids by sperm storage tubules cells with a detrimental effect on sperm storage has been reported to vary across species and breeds. In these regards, lipid peroxidation may produce toxic substances that cause a decrease in fertility and short-term sperm retention due to irreversible damage in stored spermatozoa [22], and these effects may be frequent in some breeds than in others. Gebriel et al [23] reported that highly significant differences in the IgY antibody concentration in blood serum and egg yolk of breeding hens were found when compared local hen strains. These authors also reported that high levels of IgY antibodies improved fertility, hatchability, and embryo viability.

Egg shape has also been reported to be influenced by genetic factors and individual traits and is determined in the oviduct. At ovulation, the yolk mass from the largest follicle is captured by the funnel-shaped open end of the proximal oviduct, the infundibulum. Then, it descends through the oviduct where deposition of different components of the egg occurs [24]. Eggs are classified depending on their shape index into sharp (SI<72), standard (SI = 72 to 76), and round eggs (SI>76). A high discriminating power was reported for shape index because of the wide variability across eggs of different genotypes used in this research. Araucana hens were reported to lay rounder eggs than the rest of the breeds and varieties. This is one of the causes for Araucana breed clustering in a different group from the rest of the Mediterranean breeds in the cladogram (Figure 5).

Hatchability has been reported to be higher in standard eggs. This is because the embryo can easily change its axial orientation in the egg in the later stages of embryonic development [25]. Genetic and phenotypic correlations between shape index and eggshell dynamic stiffness, undesirable in terms of hatchability, have been reported [26]. Thus, shape index must be monitored in breeding programs when aiming to select an optimal eggshell quality and ensure good reproductive parameters. Additionally, egg shape plays a pivotal role at hatcheries since rounder eggs might be easily confounded when set in the incubator, which results in a decreased hatchability [27].

The highest discriminating power was reported for egg weight. Previous research has evidenced the existence of significant differences in egg weight and size when autochthonous hen breeds are compared to commercial strains [3,7,8]. Hence, certain genotype-related factors may be inferred to influence embryo quality and viability and they may strongly be ascribed to differences in egg weight, which in turn conditions the daily metabolic activity of the embryo during incubation [28].

The performance of chicks at hatching is associated to the nutrients provided by the yolk sac. The yolk is lighter in lighter eggs thus the lipid mobilization and lipoprotein transfer, that allow the embryo development, is reduced [29]. Other factors which can influence chick viability are body weight and feed intake, which are lower for chickens from smaller eggs [30]. By contrast, the ratio of egg weight to the eggshell surface area must also be considered. In these regards, eggshell surface is relatively lower in larger eggs which difficult the gas exchange through the shell and is detrimental for the embryo. Quality of chick and growth during the first days of life at the farm are highly affected by weight-related traits, which are dependent on hen genotype. Researchers agree that eggs of average weight (neither light or heavy) produce the best results, in terms of hatchability and fertility, in many species like hens, turkeys, ostriches, and ducks [31].

Figure 5 reports a clear diversification of the native breeds and varieties studied when hatchability and external egg characterization traits are considered to build the rules for agglomeration criteria. In this regard, Araucana’s egg group differed from the Mediterranean breeds. The geographical isolation of the Araucana breed has promoted a genetic and phenotypic drift from the rest, which in turn translates into differential characteristics of the egg and its reproductive parameters [32].

Similarities across traits in Franciscan, Partridge, and White varieties of Utrerana breed were expected since such varieties are genetically close [33]. The separation of Black Utrerana from the rest of Utrerana varieties may be worth noting. The present research suggests the Black Utrerana variety may be closer to Blue Andalusian breed than to the rest of varieties of Utrerana hen breed. This may be indicative of the hybridization between these two breeds since a high morphological resemblance is evident between individuals presenting a black plumage in both breeds. This hybridization between both breeds may have been brought about as an historically direct attempt of breeders to decrease inbreeding in the Black Utrerana variety.

Data mining cross-validation permitted to develop a pipeline to detect the potential introgression events. These events may have occurred during the incubation and multiplication process that the different populations suffered via the evaluation of their hatchability. The combined interpretation of these analyses with the information derived from the CHAID decision tree (Supplementary Figure S1) may enable issuing a reliable classification method for the individuals belonging to these avian populations. CHAID decision trees revealed shape index, egg weight, and hatchability have pivotal importance, as these variables allow the decision tree main node to branch off into other nodes. These classification methods may be valid even in the context of the comparison across many genotypes when they share phenotypic resemblance as a direct consequence of these populations stemming from a common original trunk.

As reported in Supplementary Table S2, 91 observations of Blue Andalusian breed were classified as Black Utrerana after the discriminant canonical analysis. This may be indicative of Blue Andalusian breed being more severely affected by introgression by Black Utrerana than vice versa. Furthermore, 68 and 67 observations from White Utrerana and Franciscan Utrerana were respectively classified as Partridge Utrerana. This suggests a historical lack of reproductive management and a crossbreeding between both varieties, to be partially ascribed to the endangered situation that the Utrerana breed faces and the low availability of pure animals. Intervariety introgression of Andalusian Tufted White and Black plumage varieties into Black and Partridge Utrerana breed varieties has also been evidenced (although connection with the rest of varieties occurs). The connection between the aforementioned genotypes may be based on the individuals belonging to such breeds sharing similar phaneroptics and morphological traits, due to the varieties being crossed in an attempt to decrease inbreeding.

Among the breeds considered, the Utrerana avian breed is the only one that has been genetically characterized using microsatellites and the study of the genetic structure of its four varieties [33]. Indeed, this study is a preliminary research and further analyses seeking the genetic characterization of all Spanish avian breed are necessary. However, as defined in Delgado Bermejo et al [34] a breed is a subspecies population formed by individuals showing heritable morphological, functional, and genetic characteristics which identify them with respect to other populations of the species in a sufficient way to be socioculturally and/or administratively recognized. Besides, phenotype is the sum of environment plus genotype. In this context, the method that we propose connects the genetic entity of the animals (as all of them have been determined to ascribe to a specific breed) with characteristics of other nature hence, it may evidence potential traces of introgression across breeds that have developed in southern Spain using phenomic information. This could be an agent for the development of economic exploratory alternatives reporting valuable outcomes supported by such information (morphological, reproductive, functional, or even cultural), which also define a breed.

## CONCLUSION

The combination between discriminant analysis and data mining has been proved and validated as an efficient method to correctly classify and detect the degree of introgression among nine native hen genotypes considering daily collectible traits such as egg weight and shape index (egg external traits) or fertility and hatchability, which permits in hatchery daily routine data registration. Selection towards the improvement of reproductive aptitudes becomes difficult due to the low heritability of these characters. However, this research suggests that the selection of eggs with standard weight and shape (easily selectable variables) may be a good and straightforward manner to optimize hatching results.

## Figures and Tables

**Table 1:** Number of reproductive hens (N) and observations (egg number, n) per breed and variety studied

Breed and variety	N	n
Black Andalusian Tufted	8	116
White Andalusian Tufted	8	136
Blue Andalusian	10	150
Spanish White-Faced	8	110
Black Utrerana	15	261
Franciscan Utrerana	15	212
Partridge Utrerana	15	243
White Utrerana	15	140
Araucana	4	32
Total	98	1,400

**Table 2:** Multicollinearity analysis of hatchability and external characterization-related traits of eggs

Statistic/traits	Tolerance (1-R^2^)	VIF^1)^
Eggshell b*	0.4870	2.0533
Eggshell L*	0.5314	1.8818
Hatchability	0.6153	1.6253
Fertility	0.6727	1.4865
Post-hatching mortality	0.7257	1.3781
Mortality at hatching	0.7336	1.3632
Embryonic mortality 1	0.8139	1.2286
Eggshell a*	0.8793	1.1373
Egg weight	0.9643	1.0370
Shape index	0.9742	1.0265

VIF, variance inflation factor.

1) Interpretation thumb rule: VIF≥5 (highly correlated); 1<VIF<5 (moderately correlated); VIF = 1 (not correlated).

**Table 3:** Multicollinearity analysis of hatchability and external characterization-related traits of eggs

Test of function(s)	Wilks' Lambda	Chi-square	df	Sig.
1 through 8	0.511	934.430	24	<0.001
2 through 8	0.724	450.342	14	<0.001
3 through 8	0.910	130.951	6	<0.001

**Table 4:** Results for the tests of equality of group means to test for difference in the means across sample groups once redundant variables have been removed

Variable	Wilks' Lambda	F	df1	df2	p-value	Rank
Egg weight	0.7861	47.3139	8	1,391	<0.0001	1
Shape index	0.7871	47.0209	8	1,391	<0.0001	2
Fertility	0.8076	41.4270	8	1,391	<0.0001	3
Hatchability	0.9457	9.9922	8	1,391	<0.0001	4
Embryonic mortality 1	0.9839	2.8516	8	1,391	0.0038	5
Mortality at hatching	0.9854	2.5723	8	1,391	0.0087	6
Post-hatching mortality	0.9870	2.2814	8	1,391	0.0200	7
Eggshell L*	0.9915	1.4937	8	1,391	0.1546	8
Eggshell a*	0.9932	1.1874	8	1,391	0.3029	9
Eggshell b*	0.9955	0.7938	8	1,391	0.6081	10

## SUPPLEMENTARY MATERIAL

**Supplementary Table S1.** Variable discard process depending on a VIF≥5 criterion for hatchability and external characterization-related traits of eggs in the studied breeds

**Supplementary Table S2.** Studied observations (Prior) in the present study and how they were classified (Posterior) after discriminant canonical analysis

**Supplementary Figure S1.** Data mining CHAID decision tree obtained of chi-square dissimilarity matrix.
