# Supplementary material for: Discriminant canonical analysis as a tool to determine traces of endangered native hen breed introgression through egg hatchability phenomics
Source: Anim Biosci. 2022 Nov 14;38(3):381–94. doi: 10.5713/ab.22.0163 (PMC11917415; doi:10.5713/ab.22.0163)
Supplement: Supplementary file 1 [file ab-22-0163-Supplementary-Table-S1.pdf]

Supplementary Table S1. Variable discard process depending on a VIF ≥ 5 criterion for hatchability and external characterization-related traits of eggs in the studied breeds.

Statistic	Tolerance	VIF
Major diameter	0.0058	173.2366
Shape index	0.0070	142.6506
Minor diameter	0.0093	107.9251
Embryonic mortality 2	0.0421	23.7338
Viable hatching chick	0.0446	22.4038
Fertility	0.0573	17.4537
Hatchability	0.0607	16.4817
Embryonic mortality 1	0.1063	9.4091
Egg weight	0.1507	6.6379
Eggshell b*	0.4865	2.0556
Eggshell L*	0.5300	1.8867
Mortality post-hatching	0.7194	1.3900
Eggshell a*	0.8789	1.1378
Embryonic mortality	0.0000	-
Mortality at hatching	0.0000	-

Statistic	Tolerance	VIF
Embryonic mortality 2	0.0421	23.7337
Viable hatching chick	0.0447	22.3929
Fertility	0.0573	17.4537
Hatchability	0.0607	16.4754
Embryonic mortality 1	0.1063	9.4090
Minor diameter	0.1413	7.0750
Egg weight	0.1554	6.4347
Shape index	0.4168	2.3990
Eggshell b*	0.4867	2.0547
Eggshell L*	0.5303	1.8859
Mortality post-hatching	0.7196	1.3897
Eggshell a*	0.8791	1.1375
Embryonic mortality	0.0000	-
Mortality at hatching	0.0000	-

Statistic	Tolerance	VIF
Embryonic mortality	0.0341	29.3029
Viable hatching chick	0.0447	22.3929
Fertility	0.0573	17.4537
Hatchability	0.0607	16.4754
Minor diameter	0.1413	7.0750
Egg weight	0.1554	6.4347
Shape index	0.4168	2.3990
Eggshell b*	0.4867	2.0547
Eggshell L*	0.5303	1.8859
Mortality post-hatching	0.7196	1.3897
Embryonic mortality 1	0.7938	1.2598
Eggshell a*	0.8791	1.1375
Mortality at hatching	0.0000	-

Statistic	Tolerance	VIF
Viable hatching chick	0.0577	17.3398
Hatchability	0.0607	16.4754
Minor diameter	0.1413	7.0750
Egg weight	0.1554	6.4347
Shape index	0.4168	2.3990
Mortality at hatching	0.4320	2.3149
Eggshell b*	0.4867	2.0547
Eggshell L*	0.5303	1.8859
Fertility	0.6633	1.5075
Mortality post-hatching	0.7196	1.3897
Embryonic mortality 1	0.7938	1.2598
Eggshell a*	0.8791	1.1375

Statistic	Tolerance	VIF
Minor diameter	0.1415	7.0693
Egg weight	0.1555	6.4326
Shape index	0.4171	2.3972
Eggshell b*	0.4867	2.0545
Eggshell L*	0.5305	1.8852
Hatchability	0.6152	1.6255
Fertility	0.6727	1.4866
Mortality post-hatching	0.7255	1.3783
Mortality at hatching	0.7334	1.3634
Embryonic mortality 1	0.8138	1.2288
Eggshell a*	0.8793	1.1373

Statistic	Tolerance	VIF
Eggshell b*	0.4870	2.0533
Eggshell L*	0.5314	1.8818
Hatchability	0.6153	1.6253
Fertility	0.6727	1.4865
Post-hatching mortality	0.7257	1.3781
Mortality at hatching	0.7336	1.3632
Embryonic mortality 1	0.8139	1.2286
Eggshell a*	0.8793	1.1373
Egg weight	0.9643	1.0370
Shape index	0.9742	1.0265
