# Supplementary material for: Discriminant canonical analysis as a tool to determine traces of endangered native hen breed introgression through egg hatchability phenomics
Source: Anim Biosci. 2022 Nov 14;38(3):381–94. doi: 10.5713/ab.22.0163 (PMC11917415; doi:10.5713/ab.22.0163)
Supplement: Supplementary file 2 [file ab-22-0163-Supplementary-Table-S2.pdf]

Supplementary Table S2. Studied observations (Prior) in the present study and how they were classified (Posterior) after discriminant canonical analysis.

Prior → Posterior	Observations (n)	Percentage
Black Andalusian Tufted → Black Andalusian Tufted	31	2,21%
White Andalusian Tufted → Black Utrerana	74	5,29%
Black Andalusian Tufted → Partridge Utrerana	45	3,21%
Spanish White-Faced → Spanish White-Faced	44	3,14%
White Andalusian Tufted → White Andalusian Tufted	9	0,64%
White Andalusian Tufted → Partridge Utrerana	26	1,86%
White Andalusian Tufted → Black Andalusian Tufted	8	0,57%
Black Andalusian Tufted → Black Utrerana	35	2,50%
Araucana → Araucana	30	2,14%
Spanish White-Faced → Franciscan Utrerana	10	0,71%
Spanish White-Faced → Black Utrerana	38	2,71%
White Andalusian Tufted → White Utrerana	4	0,29%
White Andalusian Tufted → Spanish White-Faced	1	0,07%
Black Utrerana → Black Utrerana	158	11,29%
Black Utrerana → White Andalusian Tufted	9	0,64%
Black Utrerana → Partridge Utrerana	30	2,14%
Black Utrerana → Franciscan Utrerana	24	1,71%
Black Utrerana → Spanish White-Faced	21	1,50%
Black Utrerana → Black Andalusian Tufted	5	0,36%
White Utrerana → Black Utrerana	34	2,43%
White Utrerana → White Andalusian Tufted	5	0,36%
White Utrerana → Partridge Utrerana	68	4,86%
White Utrerana → Spanish White-Faced	3	0,21%
White Utrerana → Franciscan Utrerana	9	0,64%
White Utrerana → Black Andalusian Tufted	9	0,64%
Franciscan Utrerana → Partridge Utrerana	67	4,79%
Franciscan Utrerana → Black Utrerana	51	3,64%
Franciscan Utrerana → Franciscan Utrerana	38	2,71%
Patridge Utrerana → Patridge Utrerana	95	6,79%
Patridge Utrerana → Black Andalusian Tufted	22	1,57%
Patridge Utrerana → Franciscan Utrerana	31	2,21%
Patridge Utrerana → Black Utrerana	54	3,86%
Patridge Utrerana → Spanish White-Faced	12	0,86%
Patridge Utrerana → White Utrerana	2	0,14%
Blue Andalusian → Spanish White-Faced	4	0,29%
Blue Andalusian → Black Utrerana	91	6,50%
Blue Andalusian → Black Andalusian Tufted	14	1,00%
Blue Andalusian → White Utrerana	1	0,07%
Spanish White-Faced → Patridge Utrerana	6	0,43%
Franciscan Utrerana → Araucana	33	2,36%
Patridge Utrerana → Araucana	20	1,43%
Spanish White-Faced → Araucana	9	0,64%
White Andalusian Tufted → Franciscan Utrerana	10	0,71%
Black Utrerana → Araucana	7	0,50%
White Utrerana → Araucana	4	0,29%
Blue Andalusian → Patridge Utrerana	21	1,50%
Franciscan Utrerana → Spanish White-Faced	9	0,64%

Prior → Posterior	Observations (n)	Percentage
Franciscan Utrerana → White Andalusian Tufted	4	0,29%
Patridge Utrerana → Blue Andalusian	3	0,21%
Franciscan Utrerana → White Utrerana	5	0,36%
Franciscan Utrerana → Black Andalusian Tufted	5	0,36%
White Andalusian Tufted → Blue Andalusian	2	0,14%
Black Utrerana → Blue Andalusian	7	0,50%
Blue Andalusian → Blue Andalusian	6	0,43%
Blue Andalusian → White Andalusian Tufted	5	0,36%
Blue Andalusian → Araucana	6	0,43%
White Utrerana → White Utrerana	7	0,50%
Patridge Utrerana → White Andalusian Tufted	4	0,29%
Black Andalusian Tufted → Franciscan Utrerana	3	0,21%
Black Andalusian Tufted → White Andalusian Tufted	2	0,14%
Spanish White-Faced → White Andalusian Tufted	2	0,14%
Blue Andalusian → Franciscan Utrerana	2	0,14%
White Andalusian Tufted → Araucana	2	0,14%
White Andalusian Tufted → Blue Andalusian	1	0,07%
Araucana → Spanish White-Faced	2	0,14%
White Utrerana → Blue Andalusian	1	0,07%
**Total**	1400	100,00%
