# Supplementary material for: Discriminant canonical analysis as a tool to determine traces of endangered native hen breed introgression through egg hatchability phenomics
Source: Anim Biosci. 2022 Nov 14;38(3):381–94. doi: 10.5713/ab.22.0163 (PMC11917415; doi:10.5713/ab.22.0163)
Supplement: Supplementary file 3 [file ab-22-0163-Supplementary-Fig-S1.pdf]

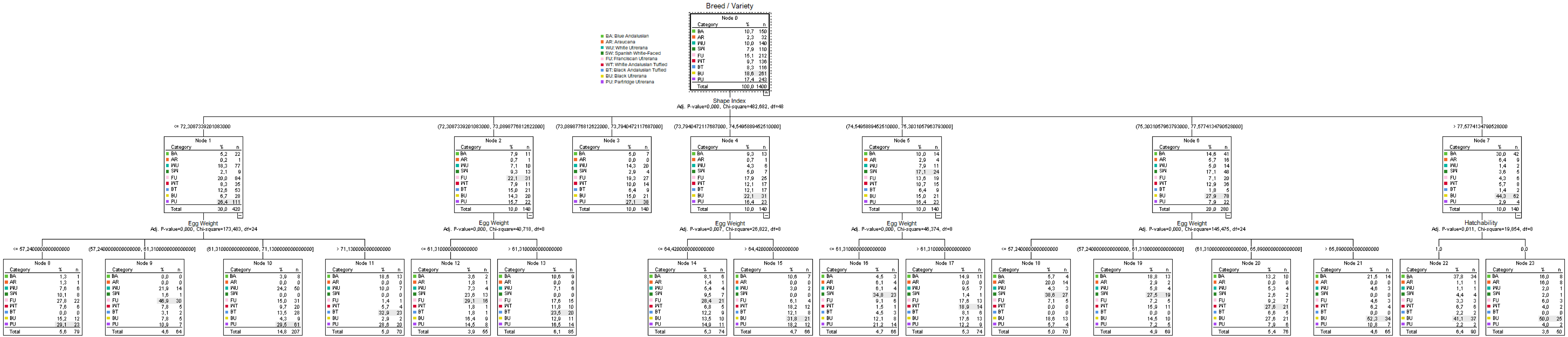
Breed / Variety
BA: Blue Andalusian
AR: Araucana
WU: White Utrerana
SW: Spanish White-Faced
FU: Franciscan Utrerana
WT: White Andalusian Tufted
BT: Black Andalusian Tufted
BU: Black Utrerana
PU: Partridge Utrerana
Node 0
Category % n
BA 10,7 150
AR 2,3 32
WU 10,0 140
SW 7,9 110
FU 15,1 212
WT 9,7 136
BT 8,3 116
BU 18,6 261
PU 17,4 243
Total 100,0 1400
Shape Index
Adj. P-value=0,000, Chi-square=482,682, df=48
<= 72,3087339201083000
(72,3087339201083000, 73,0898776812622000]
(73,0898776812622000, 73,7940472117687000]
(73,7940472117687000, 74,5495889452510000]
(74,5495889452510000, 75,3031057963793000]
(75,3031057963793000, 77,5774134790528000]
> 77,5774134790528000
Node 1
Category % n
BA 5,2 22
AR 0,2 1
WU 18,3 77
SW 2,1 9
FU 20,0 84
WT 8,3 35
BT 12,6 53
BU 6,7 28
PU 26,4 111
Total 30,0 420
Egg Weight
Adj. P-value=0,000, Chi-square=173,483, df=24
Node 2
Category % n
BA 7,9 11
AR 0,7 1
WU 7,1 10
SW 9,3 13
FU 22,1 31
WT 7,9 11
BT 15,0 21
BU 14,3 20
PU 15,7 22
Total 10,0 140
Egg Weight
Adj. P-value=0,000, Chi-square=40,718, df=8
Node 3
Category % n
BA 5,0 7
AR 0,0 0
WU 14,3 20
SW 2,9 4
FU 19,3 27
WT 10,0 14
BT 6,4 9
BU 15,0 21
PU 27,1 38
Total 10,0 140
Node 4
Category % n
BA 9,3 13
AR 0,7 1
WU 4,3 6
SW 5,0 7
FU 17,9 25
WT 12,1 17
BT 12,1 17
BU 22,1 31
PU 16,4 23
Total 10,0 140
Egg Weight
Adj. P-value=0,007, Chi-square=26,822, df=8
Node 5
Category % n
BA 10,0 14
AR 2,9 4
WU 7,9 11
SW 17,1 24
FU 13,6 19
WT 10,7 15
BT 6,4 9
BU 15,0 21
PU 16,4 23
Total 10,0 140
Egg Weight
Adj. P-value=0,000, Chi-square=46,374, df=8
Node 6
Category % n
BA 14,6 41
AR 5,7 16
WU 5,0 14
SW 17,1 48
FU 7,1 20
WT 12,9 36
BT 1,8 5
BU 27,9 78
PU 7,9 22
Total 20,0 280
Egg Weight
Adj. P-value=0,000, Chi-square=145,475, df=24
Node 7
Category % n
BA 30,0 42
AR 6,4 9
WU 1,4 2
SW 3,6 5
FU 4,3 6
WT 5,7 8
BT 1,4 2
BU 44,3 62
PU 2,9 4
Total 10,0 140
Hatchability
Adj. P-value=0,011, Chi-square=19,854, df=8
<= 57,2400000000000000
(57,2400000000000000, 61,3100000000000000]
(61,3100000000000000, 71,1300000000000000]
> 71,1300000000000000
<= 61,3100000000000000
> 61,3100000000000000
<= 64,4200000000000000
> 64,4200000000000000
<= 61,3100000000000000
> 61,3100000000000000
<= 57,2400000000000000
(57,2400000000000000, 61,3100000000000000]
(61,3100000000000000, 65,8900000000000000]
> 65,8900000000000000
1,0
0,0
Node 8
Category % n
BA 1,3 1
AR 1,3 1
WU 7,6 6
SW 10,1 8
FU 27,8 22
WT 7,6 6
BT 0,0 0
BU 15,2 12
PU 29,1 23
Total 5,6 79
Node 9
Category % n
BA 0,0 0
AR 0,0 0
WU 21,9 14
SW 1,6 1
FU 46,9 30
WT 7,8 5
BT 3,1 2
BU 7,8 5
PU 10,9 7
Total 4,6 64
Node 10
Category % n
BA 3,9 8
AR 0,0 0
WU 24,2 50
SW 0,0 0
FU 15,0 31
WT 9,7 20
BT 13,5 28
BU 4,3 9
PU 29,5 61
Total 14,8 207
Node 11
Category % n
BA 18,6 13
AR 0,0 0
WU 10,0 7
SW 0,0 0
FU 1,4 1
WT 5,7 4
BT 32,9 23
BU 2,9 2
PU 28,6 20
Total 5,0 70
Node 12
Category % n
BA 3,6 2
AR 1,8 1
WU 7,3 4
SW 23,6 13
FU 29,1 16
WT 1,8 1
BT 1,8 1
BU 16,4 9
PU 14,5 8
Total 3,9 55
Node 13
Category % n
BA 10,6 9
AR 0,0 0
WU 7,1 6
SW 0,0 0
FU 17,6 15
WT 11,8 10
BT 23,5 20
BU 12,9 11
PU 16,5 14
Total 6,1 85
Node 14
Category % n
BA 8,1 6
AR 1,4 1
WU 5,4 4
SW 9,5 7
FU 28,4 21
WT 6,8 5
BT 12,2 9
BU 13,5 10
PU 14,9 11
Total 5,3 74
Node 15
Category % n
BA 10,6 7
AR 0,0 0
WU 3,0 2
SW 0,0 0
FU 6,1 4
WT 18,2 12
BT 12,1 8
BU 31,8 21
PU 18,2 12
Total 4,7 66
Node 16
Category % n
BA 4,5 3
AR 6,1 4
WU 6,1 4
SW 34,8 23
FU 9,1 6
WT 1,5 1
BT 4,5 3
BU 12,1 8
PU 21,2 14
Total 4,7 66
Node 17
Category % n
BA 14,9 11
AR 0,0 0
WU 9,5 7
SW 1,4 1
FU 17,6 13
WT 18,9 14
BT 8,1 6
BU 17,6 13
PU 12,2 9
Total 5,3 74
Node 18
Category % n
BA 5,7 4
AR 20,0 14
WU 4,3 3
SW 38,6 27
FU 7,1 5
WT 0,0 0
BT 0,0 0
BU 18,6 13
PU 5,7 4
Total 5,0 70
Node 19
Category % n
BA 18,8 13
AR 2,9 2
WU 5,8 4
SW 27,5 19
FU 7,2 5
WT 15,9 11
BT 0,0 0
BU 14,5 10
PU 7,2 5
Total 4,9 69
Node 20
Category % n
BA 13,2 10
AR 0,0 0
WU 5,3 4
SW 2,6 2
FU 9,2 7
WT 27,6 21
BT 6,6 5
BU 27,6 21
PU 7,9 6
Total 5,4 76
Node 21
Category % n
BA 21,5 14
AR 0,0 0
WU 4,6 3
SW 0,0 0
FU 4,6 3
WT 6,2 4
BT 0,0 0
BU 52,3 34
PU 10,8 7
Total 4,6 65
Node 22
Category % n
BA 37,8 34
AR 1,1 1
WU 1,1 1
SW 4,4 4
FU 3,3 3
WT 6,7 6
BT 2,2 2
BU 41,1 37
PU 2,2 2
Total 6,4 90
Node 23
Category % n
BA 16,0 8
AR 16,0 8
WU 2,0 1
SW 2,0 1
FU 6,0 3
WT 4,0 2
BT 0,0 0
BU 50,0 25
PU 4,0 2
Total 3,6 50
